# Investigations on the Effects of Dietary Essential Oils and Different Husbandry Conditions on the Gut Ecology in Piglets after Weaning

**DOI:** 10.1155/2009/730809

**Published:** 2009-05-07

**Authors:** P. Janczyk, R. Pieper, V. Urubschurov, K. R. Wendler, W. B. Souffrant

**Affiliations:** ^1^Research Unit “Oskar Kellener”, Research Institute for the Biology of Farm Animals, Wilhelm-Stahl-Allee 2, 18196 Dummerstorf, Germany; ^2^Institute of Veterinary Anatomy, Faculty of Veterinary Medicine, Freie Universität Berlin, Koser straße 20, 14195 Berlin, Germany; ^3^Institute of Animal Nutrition, Faculty of Veterinary Medicine, Freie Universität Berlin, Bruemmer straße 34, 14195 Berlin, Germany; ^4^Delacon Biotechnik GmbH, Weissenwolff straße 14, A-4221 Steyregg, Austria

## Abstract

Essential oils (EO) are being considered as possible alternatives to in-feed antibiotic growth promoters in pig nutrition. The effects of an EO mixture consisting of limonene, eugenol and pinene (10.0, 2.0, and 4.8 mg/kg diet, resp.) on gut physiology and ecology were studied in piglets. The experiment was conducted at low (commercial farm) and high hygienic conditions (experimental farm), to elucidate interactions between EO supplementation and husbandry methods. Piglets were weaned at 28 days of age, when they were offered either a control diet (C) or C with EO. Four piglets were sacrificed in each group on day 29, 30, 33 and 39. Digesta from the third distal part of the small intestine and from the colon were sampled and analysed for pH, dry matter, lactic acid, short chain fatty acids and ammonia concentrations. Enterobacteria, enterococci, lactobacilli and yeast counts were obtained by plating. Genomic DNA was extracted from digesta and polymerase chain reaction—denaturing gradient gel electrophoresis was performed. Individual microbial communities were identified at each farm. Age affected the intestinal parameters. No effects of the EO with exception for a significant reduction in colon bacterial diversity at 39 days of age could be recorded at experimental farm.

## 1. Introduction

Weaning of piglets is associated with very severe stress. Abrupt removal of the young piglets from the dam, mixing of different litters, dietary changes (from a milk to a cereal-based diet), and contact with unknown bacteria not only result in changes in the intestinal microbial community but also suppress the immune system and increase the prevalence to intestinal pathogens. In combination with other factors, such as changes in intestinal morphology and enzyme activity [1–3], this sets the scene for the development of postweaning diarrhoea with resultant performance losses.

Therefore, prophylactic in-feed antibiotics have been used for decades to ameliorate the postweaning reduction in productivity. Since the beginning of 2006, in-feed antibiotics used in pig production have been banned in the European Union. In order to sustain high productivity in pork production, researchers have searched for alternatives such as probiotics, prebiotics, and phytobiotics which might have growth promoting effects similar to in-feed antibiotics.

Essential oils (EO) are volatile, aromatic mixtures, consisting principally of terpenes and phenylpropane derivatives. They are present in many plant tissues, where their primary function is to protect the plant against bacteria and parasites. The composition of essential oils may vary depending on species [4], geographical origin, or vegetative stage [5]. Many essential oils have strong antibacterial effects in vitro [6]. Active components of essential oils are known to have antioxidative [7] and anti-inflammatory effects [8, 9]. Feeding diets supplemented with plant extracts can influence the microflora in the digestive tract of early weaned piglets by increasing the number of lactobacilli and the ratio of lactobacilli and enterobacteria in the jejunum and caecum [10, 11]. Furthermore, positive effects on the nutrient digestibility [12, 13] and growth performance [13] in piglets have been reported.

In the present study, limonene, eugenol, and pinene were chosen to be combined in a feed additive for weaned piglets. These substances are well known for their antibacterial properties [14, 15]. They were selected because of known antioxidative [7] and anti-inflammatory [16], but also for their immunomodulatory [17] and relaxant and spasmolytic effects [18, 19]. All of these effects might be especially beneficial to piglets during the stressful weaning period.

The effect of growth promoting feed additives may depend on hygienic conditions of the farm. Only small effects might be obtained in situations with high hygienic status, while the potential for performance improvements may be more pronounced under less optimal (i.e., field) conditions. Therefore, the aim of this study was to investigate the effects of essential oils in the starter diet on development of gut ecophysiology of piglets after weaning under different hygienic conditions.

## 2. Material and Methods

### 2.1. Animals and Housing

The study comprised two experiments. One experiment was carried out at the experimental pig farm (EF) of the Research Institute for the Biology of Farm Animals Dummerstorf, Germany and the other at a commercial farm (CF). Natural light regime and humidity (50–60%) were common for both facilities. No antibiotics (for prophylactic or therapeutic reasons) were given to the animals at either facility.

Piglets of German Landrace sows in 2nd to 6th parity were used for the experiment. To reduce the genetic difference between litters, all piglets at both facilities had one father boar. Piglets were weaned at 28 days of age. The whole litter from one dam (8–11 piglets of both sexes) was allocated to a pen of 4.38 m^2^ at EF and 4.20 m^2^ at CF. Eight litters per farm were included in the experiment. The experiments were approved by the Ethical Committee of the Ministry of Nutrition, Agriculture, Forestry and Fishery Mecklenburg-Vorpommern, Germany.

### 2.2. Diets and Feeding

At each farm, four litters were fed a starter diet (C), and four other litters received the starter diet containing 0.04% of the (EO) mixture starting at weaning (28 days). Composition of the diets and calculated nutrients are shown in Table 1. The essential oil mixture contained 25 g limonene, 5 g eugenol, and 12 g pinene per kg product on organic and inorganic carriers (provided by Delacon Biotechnik GmbH; Austria). Diets and water were available ad libitum. Staffing restrictions at the commercial farm meant that it was impossible to regularly weigh the animals, and record feed intake accurately, there. Therefore, body weight (BW) of the pigs from CF was recorded only just before euthanasia, and no feed conversion ratio (FCR) was calculated. At EF, all piglets were weighed at weaning, and the FI in each pen was recorded daily to calculate the FCR.

### 2.3. Sampling and Chemical Analyses

One piglet was considered a replication unit. Four piglets per treatment group, one out of each pen, were taken at random 1, 2, 5, or 11 days after weaning (at 29, 30, 33, and 39 days of age, resp.) to provide samples. All piglets were sacrificed with an intracardial injection of T61 (Intervet, Unterschleißheim, Germany) after collecting of blood samples. Small intestine (SI) and colon were dissected. The SI was divided into three equal parts, and contents from the distal part and from the colon were collected for chemical and bacteriological analyses and genomic DNA extraction.

Intestinal contents were analysed for pH, dry matter (DM), lactate (LA), ammonia, and short chain fatty acids (SCFAs). pH was measured with an inoLab pH Level 1 meter (WTW, Weilheim, Germany). DM was determined after drying for 24 hours at 60*º*C followed by 3 hours at 105°C. For other chemical analyses, each digesta sample was diluted with water (ratio 1 : 3), homogenized and centrifuged at 4500 rpm for 10 minutes at room temperature (rt). The supernatant was used for further analyses. LA concentration was determined calorimetrically after heat precipitation with concentrated sulphur acid in the presence of calcium hydroxide. The absorbance at 565 nm of the blue-violet colour complex produced following addition of p-hydroxiphenyl was measured on Spectronic 20 Genesys spectrophotometer (Spectronic Instruments, Rochester, NY, USA) using lithium lactate solution as a standard [20]. Ammonia concentration was measured using the Conway microdiffusion assay [21]. For the measurement of SCFA in the digesta, an internal standard SCFA solution was added to the sample. The mixture was centrifuged at 4000 rpm for 20 minutes at rt. The supernatant was collected and centrifuged at 13000 rpm for 10 minutes at rt. SCFAs were then analysed using gas chromatography on GC-17A chromatograph (Shimadzu Deutschland GmbH, Duisburg, Germany) using 25 m long capillary column of 0.25 mm diameter [22].

Serum haptoglobin concentration was measured in order to obtain an objective, but indirect indication of contact with provocative antigens. A commercially available competitive enzyme immunoassay (R-Biopharm, Darmstadt, Germany), specific for porcine haptoglobin, was used for analysis. All samples were assayed in duplicate. The limit of detection for this assay was 0.033 mg/mL, and the intra- and interassay coefficients of variation were 2.8% and 5.2%, respectively.

### 2.4. Microbiological Analyses

Fresh digesta samples were homogenised, serially diluted, and plated in duplicates onto selective agars (SIFIN, Berlin, Germany). Plates for enumeration of enterobacteria (Violet-Red Bile Dextrose-agar) and enterococci (Slanetz and Bartley enterococcus selective agars) were incubated aerobically at 37°C for 24 hours. Yeasts were grown for 5 days at 37°C on Sabouraud agar. Lactobacilli (LAB) were grown on MRS agar for 72 hours at 37°C in anaerobic jars using Anaerocult A (Merck, Germany). Colony forming units (CFU) from the highest countable dilution rate of both parallels were counted, and mean results are given as log CFU/g digesta.

Total genomic DNA was extracted from the digesta samples using DNASpinKit for soil (MPBiomedicals, Heidelberg, Germany) as described by Janczyk et al. [23]. Polymerase chain reaction—denaturing gradient gel electrophoresis (PCR-DGGE) analysis—was employed in order to investigate the changes in the bacterial composition of the intestinal contents. Primer set S-D-Bact-0968-a-S-GC (forward) and S-D-Bact-1401-a-A-17 (reverse) was used [24, 25]. The PCR conditions were as described by Konstantinov et al. [26]. The amplicons of 16S rRNA genes obtained by means of PCR were then separated using DGGE as described by Janczyk et al. [23] with a denaturing gradient of 40–65%. Stained gels with SYBR Gold Nucleic Acid Gel Stain (Molecular Probes, Eugene, Oregon, USA) were exposed to UV light (AlphaDigiDoc RT, Alpha Innotech Corporation, San Leandro, California, USA) for 2 seconds and photographed with a digital camera SP-500 UZ (Olympus, Hamburg, Germany) using AlphaEaseFC Software. The DGGE fingerprints were analysed using BioNumerics software Version 6.0 (Applied Maths, Inc., Sint-Martens-Latem, Belgium).

### 2.5. Statistical Analyses

Data was analysed using multifactorial model I of ANOVA (Statistica, Tulsa, USA). Effects of age, diet, and farm and diet x farm interactions were calculated. The Tukey HSD test was used to calculate which differences caused the significant effect of the factors. All results are shown as mean values ± SD. Differences were considered significant at *P* < .05.

DGGE fingerprints were analysed by calculating the richness (number of bands in a DGGE profile), diversity (Simpson diversity index), and evenness.

Single values were further compared using ANOVA and Tukey HSD tests as mentioned above. Similarity of the DGGE profiles was calculated using the Pearson correlation, and it was visualised in a cluster by means of the unweighted pair group method with averaging (UPGMA) applying the BioNumerics.

## 3. Results

### 3.1. Performance

There were no animal losses during the experiment. Feed intake was recorded for the whole litters only at EF. Individual piglets consumed 240 ± 149 g and 200 ± 29 g daily on average in the C and EO groups, respectively. Feed conversion ratio was similar in both groups (1.9 ± 0.49 and 2.1 ± 1.20 kg/kg in C and EO, resp.)

**Figure 1:**
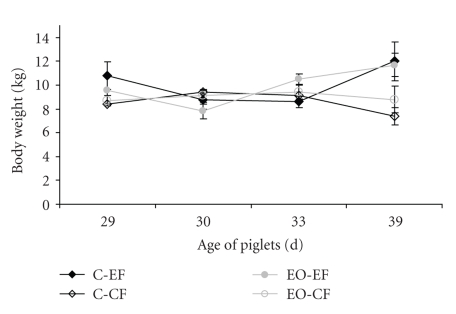
Body weight of piglets 1, 2, 5, and 11 days after weaning that were fed either a diet without (C) or with essential oils (EO) and reared at a commercial (CF) or experimental farm (EF).

Weaned piglets at the experimental farm had better growth performance than piglets at the commercial farm (Figure 1). There were no differences in body weight between the C and the EO-groups at time of sacrifice.

### 3.2. Chemical Analyses

SCFA, LA, and ammonia concentrations did not differ between C and EO at either farm but were affected by the age. The changes were observed already two days after weaning. The concentration of SCFA in colon increased from 50.8 ± 14.51 at 29 days to 86.8 ± 18.73 at 30 days and further to 106.8 ± 19.50 mmol/L at 39days (*P* < .001). LA concentration in distal SI increased from 4.7 ± 2.21 at 29 days to 24.6 ± 15.24 at 30 days and 30.9 ± 18.87 mmol/L at 39 days (*P* = .004). Ammonia concentrations in distal SI and in colon decreased from 7.4 ± 5.36 and 22.6 ± 6.90 at 29 days to 5.1 ± 1.42 and 16.6 ± 3.88 at 30 days and further to 3.5 ± 1.45 and 15.7 ± 3.80 mmol/L at 39 days, respectively (*P* = .003 and *P* < .001). These changes were accompanied by pH changes in the distal SI (7.4 ± 0.29 at 29 days, 6.8 ± 0.29 at 30 days, and 6.7 ± 0.29 at 39 days, *P* < .001) and in colon (6.6 ± 0.22 at 29 days, 6.2 ± 0.26 at 30 days, and 5.9 ± 0.27 at 39 days, *P* < .001).

Haptoglobin concentration measured in the serum (group-independent) was 1.7 ± 1.73 at EF, while it was 2.7 ± 1.95 ng/mL at CF (*P* < .001).

### 3.3. Microbiological Analyses

**Figure 2:**
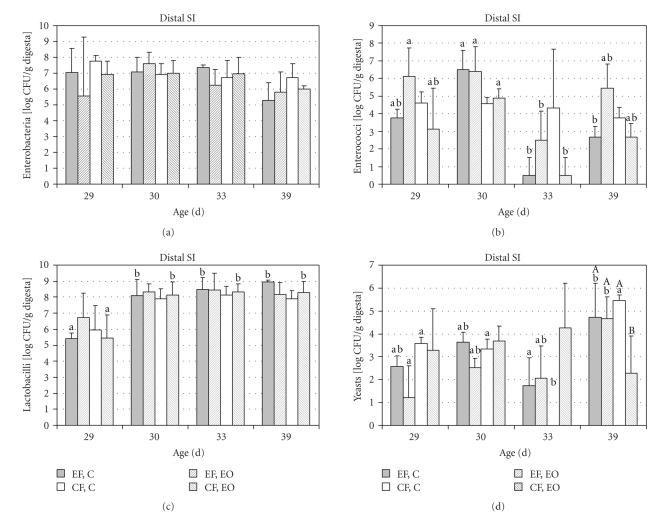
Bacterial groups and yeasts (log CFU/g) cultivated from distal small intestinal digesta collected at 29, 30 , 33, and 39 day of age from piglets fed after weaning (at 28 days) a starter diet (C) with 0.04% of essential oils mixture containing limonene, pinene and eugenol. The piglets were reared either at an experimental (EF) of commercial farm (CF). The significant differences (*P* < .05) between days within one group are marked with different small letters (a, b), and the differences between groups on one day are marked with capital letters (A, B). For clarity, only columns that differ are marked.

**Figure 3:**
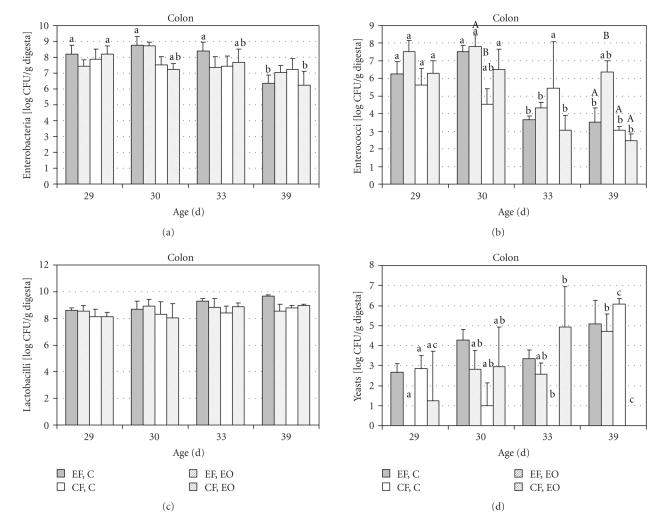
Bacterial groups and yeasts (log CFU/g) cultivated from colon digesta collected at 29, 30, 33, and 39 days of age from piglets fed after weaning (at 28 days) a starter diet (C) with 0.04% of essential oils mixture containing limonene, pinene, and eugenol. The piglets were reared either at an experimental (EF) of commercial farm (CF). The significant differences (*P* < .05) between days within one group are marked with different small letters (a, b), and the differences between groups on one day are marked with capital letters (A, B). For clarity, only columns that differ are marked.

**Figure 4:**
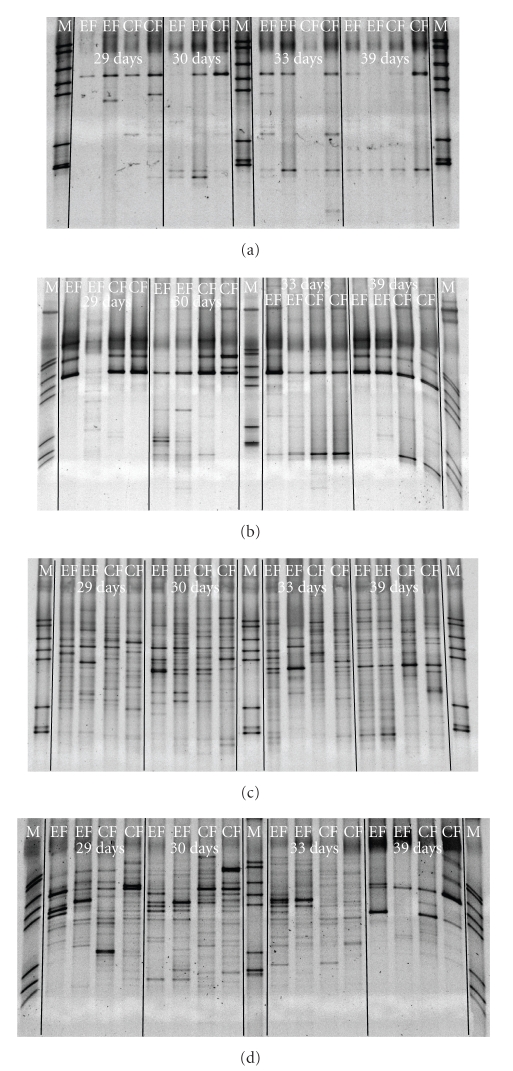
Representative images of denaturing gradient gel electrophoresis fingerprints of amplicons of bacterial V6–V8 fragments of 16S rRNA gene obtained from distal small intestinal (a, b) and colonic (c, d) digesta of piglets fed after weaning a starter diet (a, c) with 0.04% of essential oils mixture containing limonene, pinene, and eugenol (b, d).

The bacterial group and yeast counts in distal SI and in colon are presented in Figures 2, 3 and 4. Enterobacteria numbers in the intestinal digesta decreased with age (at 39 days) at both farms (*P* = .066 for distal SI, *P* < .001 for colon), with no effect of diet. Enterococci counts in the colon were influenced (*P* < .001) by farm, age, and diet, while only farm was important for enterococci counts in small intestine. At EF, their number in the gut of weaned piglets was drastically reduced at 33 days and partially recovered at 39 days in the control group, whereas at CF, this phenomenon was observed in the essential oil group. Nevertheless, enterococci counts remained low at 33 days. Small intestinal lactobacilli counts were low at 29 days and increased at 30 days. No effect of diet was observed on bacterial counts in small and large intestine up to 39 days. Yeasts counts in the intestinal digesta increased with age and were also affected by diet and farm (*P* < .01).

### 3.4. Denaturing Gradient Gel Electrophoresis

**Figure 5:**
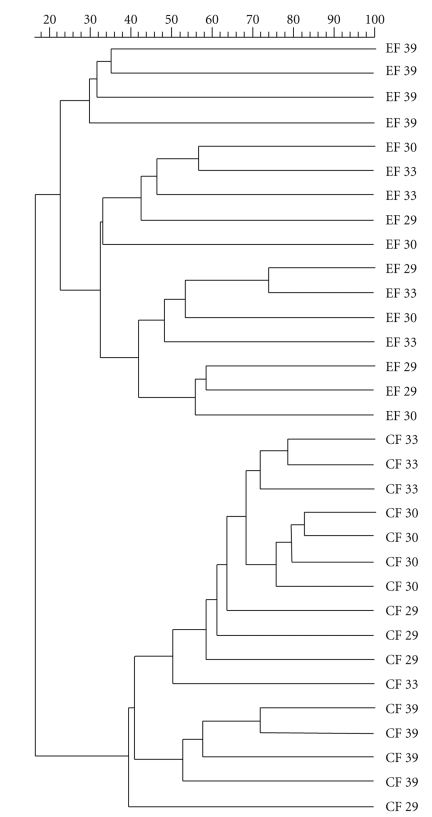
Cluster of denaturing gradient gel electrophoresis fingerprints of amplicons of bacterial V6–V8 fragments of 16S rRNA gene obtained from colon digesta of piglets fed after weaning starter diet with 0.04% of essential oils mixture containing limonene, pinene, and eugenol. Similarity was calculated using Pearson coefficient, and the cluster was formed by means of unweighted pair group method with averaging. EF—experimental farm, CR—commercial farm, 29, 30, 33, 39—age of piglets (d) corresponding to 1, 2, 5, and 11 days after weaning, respectively. Upper bar shows the distance of similarity in %.

Analysis of bacterial community composition by means of 16S rRNA PCR-DGGE revealed changes in the bacterial community with age (Figure 4). Whereas the upper bands in the gels from distal SI could be observed in all animals; the bands from the lower part of the gels could be observed first from 30 days, both in CF and EF. No differences in the DGGE patterns between C and EO in distal SI could be observed. In colon, however, already the visual analysis of the DGGE profiles revealed a difference between C and EO at 39 days, when fewer bands could be observed in the EO at either farm in comparison to the C from respective farm. The calculation of richness revealed significant differences between C and EO at 39 days (EF: 21 ± 3.8 and 5 ± 1.7, *P* < .001; CF: 16 ± 2.6 and 7 ± 1.4, *P* < .01 in C and EO, resp.). This observation was confirmed by clustering of the DGGE profiles of the bacterial 16S rRNA gene amplicons from colon digesta which showed distinct clustering of the profiles from piglets at 39 days, independent from the farm. Moreover, the clustering of the profiles using Pearson similarity showed differences between farms (Figure 5).

The Simpson diversity index of the DGGE profiles from the colon bacteria decreased in the EO group at 39 days when compared to C, however, the difference was significant only for EF (EF: 13.1 ± 1.62 and 4.6 ± 2.10, *P* < .01; CF: 8.6 ± 1.28 and 4.4 ± 0.81, *P* > .05, resp.). The calculated evenness for the DGGE fingerprints was higher in the EO than in the C group (in the range of 0.7–1.0 in C and 0.6–0.7 in EO for the investigated time points at either farm, *P* < .001). Single diet and age effects could be statistically proven only for the colon bacterial profiles and no difference was observed for ileum digesta samples.

## 4. Discussion

In this study, a mixture of limonene, eugenol, and pinene was fed to weaned piglets but resulted in no effect on piglet production parameters. Performance data, however, were only recorded as descriptive parameters in order to ensure the animals were in overall good condition. Therefore, no final conclusion on the effects of the tested essential oil mixture on piglet performance can be drawn.

The choice of conditions (in this case, a farm), where experiments are conducted, can be of importance for observed results. No effects of diet containing in-feed antimicrobials on intestinal microbiota could be observed when an experiment was conducted at a farm with high and constant hygienic conditions [27]. Therefore, the present study was conducted in parallel at two farms, assuming a commercial farm would have lower hygienic conditions because of the lack of a high grade sanitary regime. The hygienic status of the farms was defined “high” (EF) or “low” (CF) according to observations of the farms before the experiment. In the modern EF, built in 2001, five technicians take care of 70 sows. Pens have a plastic grid floor with a solid heated plate in the middle, and they are cleaned 3 times a week with a disinfectant when occupied. By contrast, the more than 45-years-old CF keeps 600 sows and employs 6 technical staff. Pens are provided with concrete floor and when occupied, the faeces are removed from the pens, but cleaning with disinfection only happens when pens are empty. The hypothesis that CF had lower hygienic status was confirmed by measurement of pig serum haptoglobin concentrations. Haptoglobin serum concentration increases in pigs as a result of sequential (although subclinical) infections [28]. As there were no clinical signs of infections in the herd, a higher bacterial and/or viral load at CF could be the reason for the observed results.

Cereal-based starter diets usually contain high amounts of complex plant carbohydrates compared to sow milk. This requires adaptation of intestinal bacteria from fermentation of lactose towards a broad range of carbohydrates. Lactobacilli adapt rapidly to such changes after abrupt decrease in their numbers directly after weaning [23]. In the present study, their numbers in the SIs increased 2 days after weaning. As the main fermentation product of lactobacilli is lactic acid, this may be an explanation for the increase of lactic acid concentration in small intestine.

An increase in lactobacilli and the ratio of lactobacilli and enterobacteria in the jejunum and caecum of early weaned pigs, respectively, has been reported at dietary application on carvacrol, cinnamaldehyde, and capsicum oleoresin [10, 11]. Such an effect, however, could not be found in the present study.

Increased flow of indigestible carbohydrates to the colon results in higher production of SCFA [29]. SCFA in colon increased after weaning, indirectly showing increased feed intake. Ammonia concentration decreased as a result of the higher amounts of starch and other indigestible carbohydrates entering the large intestine, indicating less protein breakdown by indigenous microbiota. These findings correspond with the observed pH values in both parts of the GIT. All these results confirm earlier observations [27, 30–33].

A molecular approach of 16S rRNA PCR-DGGE was applied in this study to investigate the changes in the microbial community. Simpson diversity index (*S*_1_/*D*) and evenness (*E*_1_/*D*) were adapted and calculated for objective comparison of the DGGE fingerprints' richness (number of bands in a sample). Simpson diversity index is one of the most meaningful and robust diversity measures, and it captures the variance of the species abundance distribution. Its value rises when the assemblage becomes more even. Evenness is a measure of how similar the species are in their abundances and it rises when species are more equally abundant in an assemblage. This parameter is independent from the species richness [34]. At the end of the study, the richness and the diversity of the DGGE profiles were lower in the EO than in C at either farm, but significant reduction was recorded only at EF. Bacteria sensitive to active components might have disappeared or their abundance was reduced below the detection limit of the method (being 10^9^ cells per g, assuming a total of 10^11^ cells in one g of digesta [35]) allowing the remaining bacterial groups a more equal development (giving higher evenness). The reduction in the diversity of the colonic microbial population may result in increased susceptibility to diarrhoea of different origin (pathogens, toxins, fatty acids imbalance, etc.); on the other hand, potentially pathogenic bacteria may have also disappeared from the community, as it was recorded for thymol [36]. As we did not sequence the bands in this study, no conclusion can be drawn on which species were affected by the oils mixture and this could be a matter for further studies.

Interestingly, the microbiota in the intestine of the piglets from CF seemed to be more stable and less affected by the EO indicating the importance of the farm. Indeed, the DGGE fingerprints could be clustered individually for each farm, and differences in the intestinal microbial community at each farm could be confirmed. Nevertheless, the general lack of effects of the essential oils on bacterial counts was observed at both farms. However, essential oils reduced the enterococci counts at the commercial farm which was probably depended on the different bacterial loads and on the different initial compositions of the bacterial populations.

Many essential oils have strong antibacterial effects in vitro [6]. However, in vivo results often do not correspond to the findings in vitro [37, 38]. The reasons for this are unclear, possibly relating to factors such as adsorption time, transit time, or interactions with other digesta components. Sads and Bilkei [39] and Molnar and Bilkei [40] observed positive effects of feeding high levels of essential oils in pig diet (60–180 mg carvacrol and 55–165 mg thymol in kg diet), whereas others observed no effects of feeding low levels of phytogenic products [10, 41, 42]. In a previous study, it was shown that high dosage of thymol (10 g in kg diet) affected intestinal microbiota in vivo [36]. The dosage applied in the present study was chosen based on concentrations of essential oils commonly used in feed industry and scientific studies [10], which would be economically feasible, and, therefore, the essential oil content (16.8 mg essential oils per kg diet) was much lower than in the study of Janczyk et al. [36]. It, therefore, cannot be excluded that certain effects may have occurred at higher dosage of the tested essential oil mixture.

The main aim of the study was to investigate if the tested essential oils provided in economically feasible concentrations had any effects on intestinal parameters in weaned piglets kept under different farm conditions. Individual intestinal bacterial communities were observed at each farm. Limonene, pinene, and eugenol had no overall effect on weaned piglets performance or the gut parameters. However, a late effect of the tested oils could be observed on colon bacterial population composition, and farm conditions seemed to play a role in the observed changes.

## Figures and Tables

**Table 1:** Composition of the control and experimental starter diets.

Ingredients	%	Calculated nutrients	g/kg (as fed basis)
Barley meal	30.0	DM	888
Wheat meal	29.7	CP	191
PEF (44% starch)	5.0	Ash	55
Whey powder	8.0	Crude fibre	34
Wheat bran	2.5	Crude fat	50
Soycomil (soy concentrate)	4.0	Starch + sugar	455
Maize starch^1^	4.0	Lysine	12.5
Potato protein, purified	5.0	Ileal digestable lysine	11.0
Maize gluten meal	2.2	Methionine	4.4
Sunflower meal	2.5	Ileal digestable methionine methionine	4.0
Limestone	1.02	Methionine+Cysteine	7.8
Mono calcium phosphate	0.78	Ileal digestable Met+Cys	6.6
Trace min.-vit. Premix^2^	0.4	Tryptophan	2.5
Methionine (99%)	0.11	Ileal digestable tryptophan	2.1
L-lysine-HCl (79%)	0.34	Threonine	8.0
Tryptophan (99%)	0.031	Ileal digestable threonine	6.5
Threonine (98%)	0.03	Ca	7.2
Palm oil + soybean oil	3.1	Total P	6.1
Molasses	1.009	Digestible P	3.65
NaCl	0.28	Na	2.5
		K	8.5
		Cl	6.7
		Cu, mg	20
		Zn, mg	90
			
		Limonene^3^	0.010
		Eugenol^3^	0.002
		Pinene^3^	0.0048

Total	100.00	NE_f_, MJ/kg	10.0

^1^Maiz starch was reduced to 3.96% in EO; 0.04% of essential oil mixture was added instead.

^2^This trace mineral-vitamin premix (0.4%) supplies per kg diet as follows: vit. A 1750 IU, vit. D_3_ 200 IU, vit. E 11 IU, vit. K_1_ 0.5 mg, vit. B_1_ 1.0 mg, vit. B_2_ 4 mg, d-pantothenic acid 9 mg, niacin 12.5 mg (available), biotin 50 *μ*g, vit. B_12_ 15 *μ*g, folic acid 0.3 mg, vit. B_6_ 1.5 mg, choline 400 mg, Fe 80 mg, Zn 54 mg, Mn 30 mg, Co 0.15 mg, I 0.14 mg, Se 0.25 mg, antioxidants (E310,320,321) 50 mg, and maize starch as carrier.

^3^Essential oils in the EO.
